# Tendencies, variability and persistence of sea surface temperature anomalies

**DOI:** 10.1038/s41598-020-64785-9

**Published:** 2020-05-14

**Authors:** Claire E. Bulgin, Christopher J. Merchant, David Ferreira

**Affiliations:** 1 University of Reading, Department of Meteorology, Reading, RG6 6AL UK; 2 National Centre for Earth Observation, Leicester, UK

**Keywords:** Physical oceanography, Physical oceanography

## Abstract

Quantifying global trends and variability in sea surface temperature (SST) is of fundamental importance to understanding changes in the Earth’s climate. One approach to observing SST is via remote sensing. Here we use a 37-year gap-filled, daily-mean analysis of satellite SSTs to quantify SST trends, variability and persistence between 1981–2018. The global mean warming trend is 0.09 K per decade globally, with 95% of local trends being between −0.1 K and + 0.35 K. Excluding perennial sea-ice regions, the mean warming trend is 0.11 K per decade. After removing the long-term trend we calculate the SST power spectra over different time periods. The maximum variance in the SST power spectra in the equatorial Pacific is 1.9 K^2^ on 1–5 year timescales, dominated by ENSO processes. In western boundary currents characterised by an intense mesoscale activity, SST power on sub-annual timescales dominates, with a maximum variance of 4.9 K^2^. Persistence timescales tend to be shorter in the summer hemisphere due to the shallower mixed layer. The median short-term persistence length is 11–14 days, found over 71–79% of the global ocean area, with seasonal variations. The mean global correlation between monthly SST anomalies with a three-month time-lag is 0.35, with statistically significant correlations over 54.0% of the global oceans, and notably in the northern and equatorial Pacific, and the sub-polar gyre south of Greenland. At six months, the mean global SST anomaly correlation falls to 0.18. The satellite data record enables the detailed characterisation of temporal changes in SST over almost four decades.

## Introduction

Among the geophysical variables that need to be quantified to understand the Earth’s climatic and biogeochemical systems, sea surface temperature (SST) is crucial^1^, playing important roles in global lateral energy transport, radiative and turbulent air-sea energy exchange, absorption of anthropogenic greenhouse gases in the ocean, modification of the atmospheric boundary layer, the global water cycle, and many other global and regional processes. For this reason, efforts to estimate the spatio-temporal progression of global SSTs have been sustained, using many approaches, one of which is Earth observation (remote sensing). A global, gap-free, 37-year timeseries of SST with feature resolution ~20 km derived from satellite infrared observations^2^ is used in this paper to quantify aspects of SST trends, variability and persistence.

This dataset is of importance in evaluating SST changes related to climate over the satellite data period due to its length (37 years) and spatial resolution (0.05 degrees). The dataset is strengthened with respect to other analysis products by the inclusion of data from the Along-Track Scanning Radiometer instruments, designed specifically to retrieve surface temperature rather than for more general meteorological applications. This dataset is to the maximum degree possible independent of *in-situ* observations^2^. Being unique in this regard, the dataset is complimentary to other analyses and useful for applications such as model verification and development^3^. An uncertainty estimate is provided for each datum quantifying the spatial and temporal variations of uncertainty^2^. The ESA CCI SST products validate well against Argo match-ups, with lower standard deviation of the SST differences than for other reanalysis products, including the Reynolds AVHRR optimal interpolation^4^.

In the remainder of this introduction, we summarise some aspects of SST dynamics relevant to the results which then follow. The dominant variability of SST is seasonal. The climatological annual temperature range varies geographically from essentially zero to >15 °C, but there is also a multi-decadal tendency to warmer SSTs because of greenhouse gas forcing of the climate system. This paper first discusses the trends in SST anomalies (SSTA) over the length of the dataset and then describes the variability and persistence of SST anomalies after the climatological seasonal signal and warming trend are removed. In other words, in the second part of the paper we quantify and discuss features of de-trended SST anomalies.

SSTA variability arises from a number of mechanisms with differing timescales. Inter-annual SSTA variability at a given location tends to be greater for some months of the year than for others, and this annual phasing of inter-annual variability in turn differs from place to place^5^. This is partly linked to the El Nino Southern Oscillation (ENSO), which is the largest mode of SSTA variability involving remote teleconnections mediated by ENSO influences on atmospheric circulation. ENSO SSTA variance is smallest in boreal summer and largest in winter^6^. In turn, ENSO state influences SSTA persistence in the Indian Ocean, where SSTA variability is comparatively small^7^. Between the Indian Ocean and central Pacific, SSTA variability in the seas around Indonesia and the western Pacific is smaller than in the central and eastern Pacific where ENSO-related amplitudes are largest. Nonetheless, variability in this Indonesian warm pool region is an important driver of atmosphere-ocean processes^8^.

The annual phasing of inter-annual SSTA variability in mid-latitudes is strongly modulated by seasonal cycles of mixed layer depth (MLD), which are driven by changes round the year in insolation and mixing forced by surface wind stress. The shallower MLD of summer periods represents a smaller effective heat capacity with correspondingly larger SSTA sensitivity to variability in atmospheric forcing. Regions characterised by a strong seasonal variation in MLD may also display the phenomenon of re-emergence of winter-to-winter SSTA^9–13^. Winter SST anomalies persist at depth during the summer months below the shallow mixed layer, reemerging in the SSTA the following winter as the summer seasonal thermocline is eroded. In the tropics, SSTA re-emergence is strongly coupled with recurrence of atmospheric drivers, predominantly wind stress^9^, and there is evidence that atmospheric drivers can also affect inter-annual variations in mid-latitude re-emergence^14^. Regional studies have demonstrated the independence of the re-emergence signal from ENSO variability^15^ with the strongest re-emergence signals in the NW Pacific, NW Atlantic and Southern Ocean^16–18^. Most studies focus on co-located re-emergence (in the same location from year-to-year), whilst a few have demonstrated the plausibility of remote re-emergence by tracking the subsurface movement of water masses over the intervening period^16,19^.

SST modes and variability are strongly coupled with the atmospheric circulation. Western boundary currents (WBCs) are turbulent areas of water characterised by strong ocean-atmosphere interaction, poleward heat transport and ocean-atmosphere heat exchange^20^. The two northern hemisphere WBCs are the Gulf Stream (Atlantic) and the Kuroshio current (Pacific), the poleward extent of which are controlled by the location of the frontal zone in which they meet the sub-polar gyres^20^. In the southern hemisphere the poleward flowing Brazilian current meets the equatorial flowing Falklands current in the southern Atlantic between 35–40°S (Brazil-Falklands Confluence) in a region characterised by SST fronts and extensive mixing^21^. The second WBC in the southern hemisphere is the Agulhas current, which follows the west African coast before heading seawards in a south-westerly direction upon reaching the southern tip of Africa. South of Cape Town at 35°S the Agulhas retroflection occurs in the subtropical convergence zone. The return current then propagates east at this latitude with large eddies shed in the region of retroflection^22,23^. Warm water also travels polewards in the East Australia Current along the Great Barrier Reef before deflecting towards New Zealand at 35°S^24^.

Rapidly fluctuating SSTAs are not confined to coastal regions but also arise from dynamical features such as Kelvin waves and Tropical Instability Waves (TIWs) in the equatorial Pacific. The oceanic Kelvin wave propagation cycle in the Pacific is ~70 days. A relaxing of the easterly trade winds drives downwelling Kelvin waves which propagate eastward in the equatorial Pacific. These depress the usually shallow thermocline and transport warmer waters towards the Ecuador and Peruvian coast^25–28^. Kelvin waves are strongest under El Nino conditions and weakest in La Nina^27^. TIWs propagate westward from the South American coast with a periodicity of 15–40 days. Variability in the strength of TIWs is coupled with both seasonal and ENSO cycles and TIWs can be modulated by Kelvin waves^29^.

Some regions of the global ocean are of interest because they exhibit stronger signals in SST variability, and we specifically look at the SST trends in these areas in the results section. These include (1) the Atlantic Meridional Variability (AMV) region in the North Atlantic. Here there is typically a strong signal in the SST anomaly on decadal timescales, which has been linked to climate impacts such as temperature and rainfall patterns, hurricane activity and changes in sea level^30^. (2) The NINO 3.4 region is defined as latitudes between 5 S–5 N and longitudes of 120–170 W and is commonly used to classify ENSO events. Sustained SST anomalies of +/−0.4 K in this region over six months are indicative of El Nino or La Nina events respectively^31^. (3) The North-East Pacific between 45–55 N and 130–150 W is of interest due to the significant warm anomaly in the SST seen between 2014–2016, which had impacts on fisheries and regional weather^32^.

The purpose of this paper is to quantify SSTA trends, variability and persistence over the last four decades using the gap-filled, daily-mean SST values of the European Space Agency (ESA) Climate Change Initiative (CCI) SST analysis^2^, further described in the section on Methods. The remainder of the paper is organised as follows: within the results section we first discuss SSTA trends, then SSTA variability and then finally SSTA persistence. We then provide a discussion of our findings including evidence for SST re-emergence and an analysis of the uncertainties in the SST data and trend fitting. A detailed description of the methods used can be found at the end of this paper.

## Results

### SST Trends

**Figure 1:**
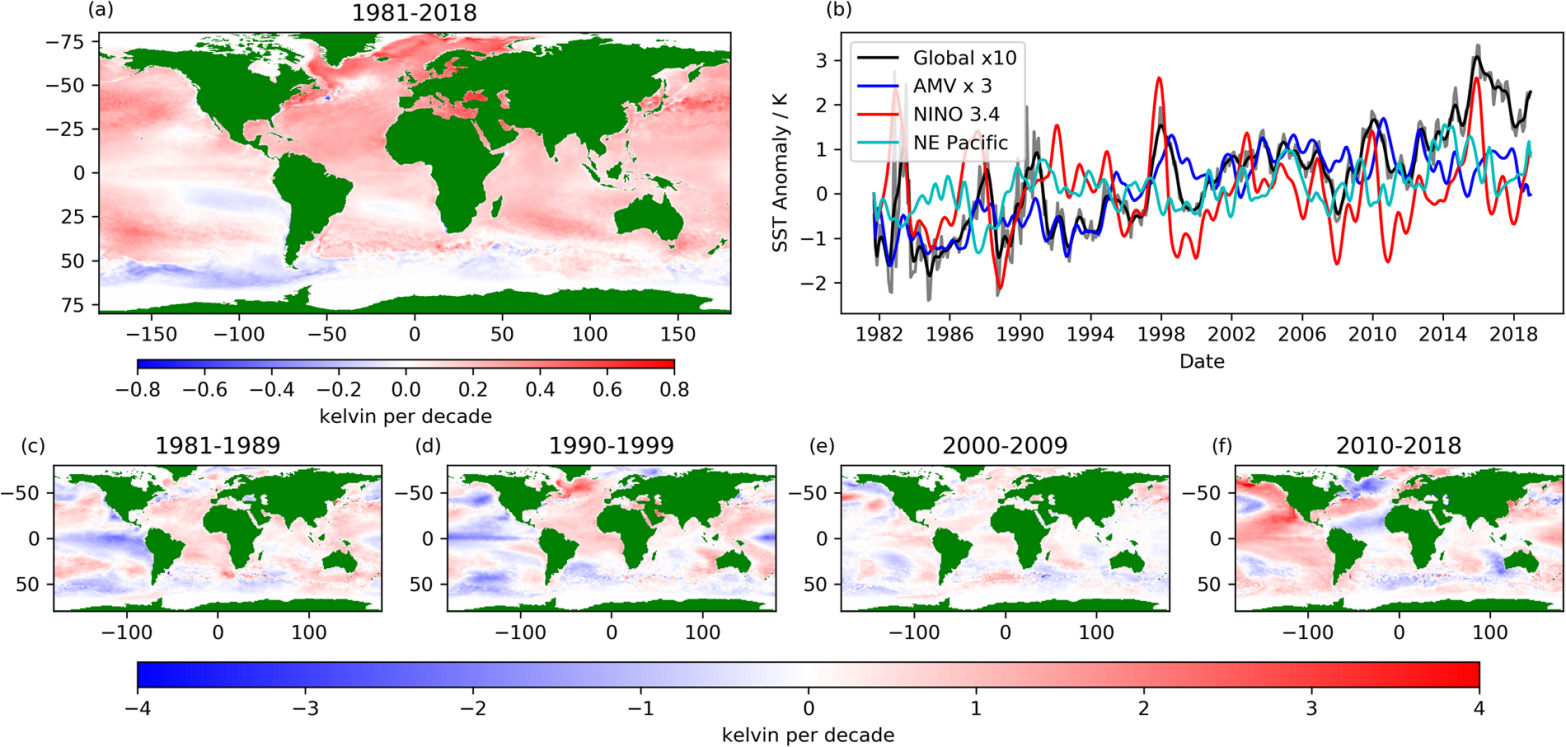
(**a**) Global sea-surface temperature anomaly (SSTA) trends between 1981–2018 relative to a climatology between 1982–2010 at 0.25 degree resolution. (**b**) Timeseries of SSTA between 1981–2018 for the global data (raw data in grey, smoothed data in black, multiplied by a factor of 10), the Atlantic Meridional Variability Region [0–65 N, 75–7.5 W^30^ (blue, multiplied by a factor of 3), the Nino 3.4 region [5N-5S, 170–120 W^31^ (red) and the NE Pacific warm waters [32–54 N, 165–138 W^32^ (cyan). Decadal tendencies (degrees kelvin per decade) calculated from data for (**c**) 1981–1989, (**d**) 1990–1999, (**e**) 2000–2009 and (**f**) 2010–2018 are at one degree resolution. Note that the colour scale is different (+/−4 K) for decadal panels (c–f) than for the full-period figure (a,+/−0.8 K).

We first consider trends in SSTA between 1981–2018 with reference to the mean SST during the period 1982–2010 (Fig. 1a). The global mean area-weighted trend in SST is 0.09 ± 0.006 K per decade including perennial sea-ice regions and 0.11 ± 0.008 K per decade excluding these. Perennial sea-ice regions are those locations affected by seasonal sea-ice for part or all of the data record. For reference, all sea-ice regions (both permanent and seasonal) are identified in Figs. 3–6 by light grey shading. This warming trend is consistent with other estimates made during the satellite era (1979–2012) cited within the Intergovernmental Panel on Climate Change (IPCC) Assessment Review 5^33,34^. The largest positive SST anomalies were observed in 2016–2017 (global SSTA time series, Fig. 1b). The tendency of SSTA between 1981–2018 is for a positive temperature trend at almost all locations across the global oceans. An exception is the Southern Ocean (south of about 50°S) where temperatures have cooled or remained nearly constant at most longitudes. This cooling trend persists through the 1980’s-2000’s, consistent with SST analysis products constrained by *in-situ* data (all be it sparsely in the region of interest)^35^ and only during the last decade is a warming trend seen here.

The strongest warming trends are in the Northern Hemisphere close to the Arctic sea-ice boundary, in the Mediterranean, in the Black Sea and along the northern edge of the Northern Hemisphere western boundary currents (Gulf Stream and Kuroshio Extension). There are also some poleward warming trends along the southern hemisphere boundary currents (Brazilian-Falkland Confluence and Agulhas current). The localised cooling trend just south of the Gulf Stream in Fig. 1a corresponds to a localised region of increased SST anomaly variability at the edge of the Grand Banks where the cold Labrador Current and warm Gulf Stream meet.

Figure 1b shows the SSTA timeseries throughout the full data record for three regions of interest: (1) The Atlantic Meridional Variability (AMV) region^30^, (2) The NINO 3.4 region^31^ and (3) the NE Pacific where a persistent warm anomaly was observed within the past decade^32^. The AMV region shows a step change in the SSTA centred on 1995, corresponding to the change to a positive phase of the AMV^30^. Changes in the AMV are coupled with the North Atlantic Oscillation and Atlantic Meridional Overturning Circulation (AMOC), with a positive phase of the AMV associated with warmer SSTs^30^. SSTA fluctuations in the Nino 3.4 region have a range of ~3–5 K, with positive SSTA’s associated with El-Nino events. Stronger ENSO events (eg. 1982–1983, 1997–1998 and 2015–2016^36^) produce the largest positive SSTA’s, with a correlation of 0.46 between the Nino 3.4 SST and the global mean detrended SST. The NE Pacific warm anomaly of 2014–2016 is markedly stronger (in this location) than any previous period in the dataset and is attributed to strong, persistent, positive sea-level pressure anomalies^32^.

There is considerable inter-decadal variation in within-decade temperature tendencies (Fig. 1c–f), which is particularly notable in the sub-Greenland gyre. There was a strong warming (max 3.1 K per decade) in the 1990’s and there has been a strong cooling (min −2.5 K per decade) after 2010. SSTA variability in this region has been linked to the AMOC, which was moving into a positive phase in the 1990’s (warming trend in SSTAs), peaking in the 2000’s and then declining in the 2010’s (cooling trend in SSTAs)^37–40^. It is also consistent with the subsurface temperature trends (warming from the mid-80’s to 2006 and cooling thereafter), with a time-lag between subsurface temperatures and subsequent surface temperature signals^41^. To the south of Greenland (50 N, 30 W) we see a region of neutral temperature trends, in the inter-gyre region between the sub-polar and sub-tropical gyres. Decadal temperature trends here are consistent in sign with the sub-polar gyre, but much smaller in magnitude with an overall neutral trend between 1981–2018, also seen *in-situ* data records^37^. South of the Gulf Stream, a localised cooling trend (40°N, 60°W) is observed in the 1990’s, indicative of the northward shift in the Gulf Stream with a return to a positive phase of the North Atlantic Oscillation (NAO)^42,43^ (Fig. 1d). In the 2010’s we observe a distinctive tripole signature in the SSTA trends of the North Atlantic, associated with the mixed layer response to the NAO^44^. Variability in this SST signal occurs on a variety of timescales (both inter-annual and decadal)^45,46^, and does not align with the decadal boundaries.

The “global warming hiatus”^47,48^, is seen in Fig. 1e, referring to a decade (2000 to 2009) during which global mean SSTA changed less than in the previous or subsequent decade. Moreover, the localised trends in SSTA are in general significantly smaller than in other decades, with a standard deviation in the SSTA trends of 0.33 K in the 2000’s compared with 0.49–0.66 K for the other decades. This coincides with a period of lower ENSO variability (with a standard deviation of 0.68 in the ENSO index^36^ in the 2000’s compared with 0.91–0.96 for the other decades). The sensitivity of the global mean SST response to the ENSO signal appears to have decreased post 2000 relative to the earlier part of the time series.

The areas with the strongest warming trends during 2010–2018 are the North and East Pacific, the trend being driven by the warm anomalies observed here between 2014–2016^32,49^. The warm anomaly maximum was observed in the offshore northerly region and along the west coast of the USA^49^. Temperature anomalies in the ENSO region of the tropical, eastern Pacific exhibit decadal variability - cooling in the 1980’s, warming close to the South American west coast in the 1990’s with cooling in the equatorial waters, weak warming in the 2000’s during the hiatus period, and a strong warming signal both close the the South American coast and in equatorial waters in the 2010’s. SST anomalies in the Pacific respond to multiple processes: atmosphere-ocean interaction, ENSO, SST re-emergence and the SSTA patterns described by the Pacific Decadal Oscillation^50^.

### SST Variability

**Figure 2:**
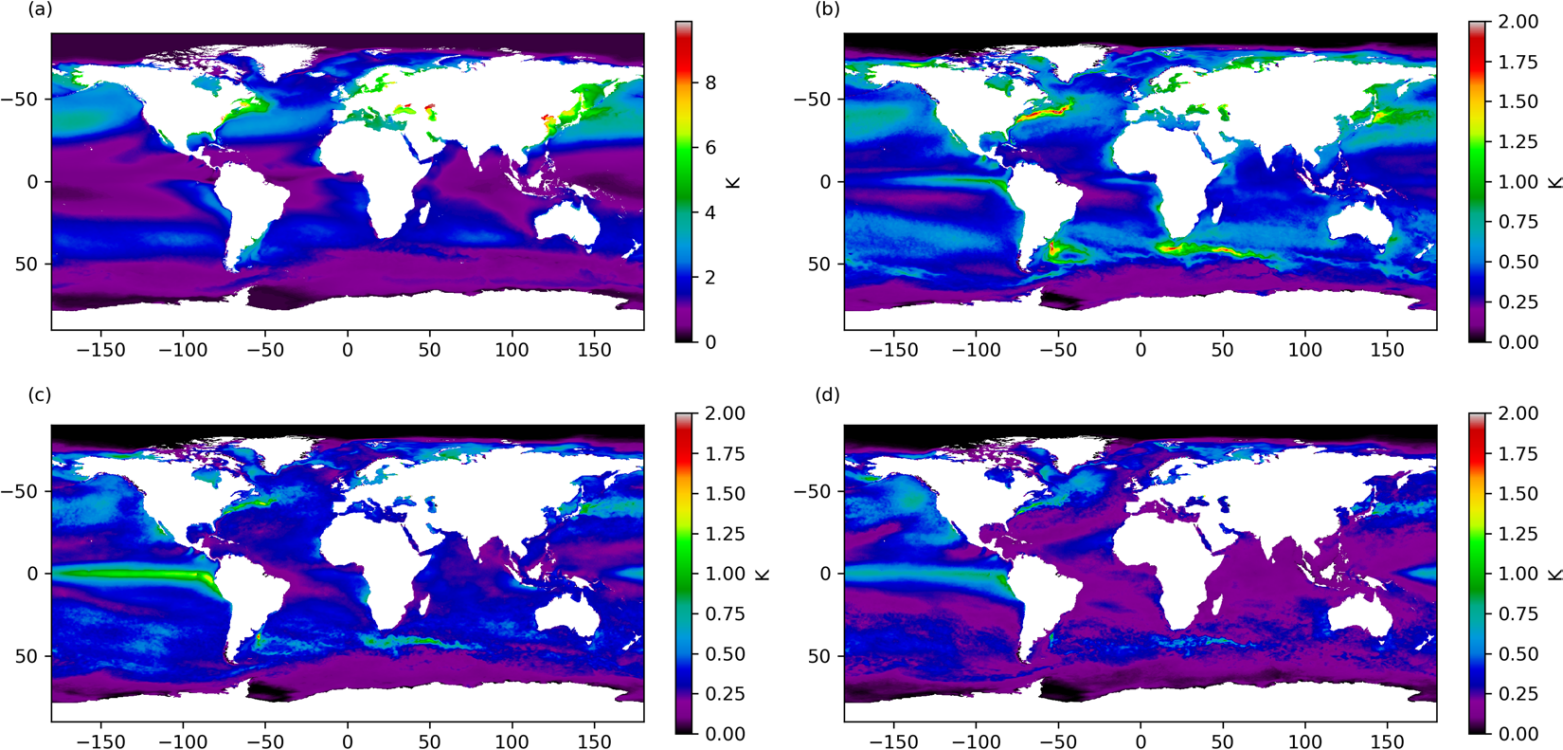
(**a**) SST variability in the monthly climatology from 1982–2010. (**b–d**) SST integrated amplitude attributable to different parts of the SST power spectrum: (**b**) sub-annual timescales, (**c**) timescales of 1–5 years, (**d**) timescales longer than 5 years.

Figure 2 shows components of SST variability. Figure 2a is the variability associated with the annual cycle, evaluated as the standard deviation of the climatological SST around the year. The annual cycle is small (~1 K) at equatorial and very high latitudes, and is at a maximum at mid-latitudes. The cycle in the northern mid-latitudes is greater than that of the southern mid-latitudes^51^, with a mean standard deviation of 3.4 K for 30–50 N vs. 1.6 K for 30–50 S. This difference in SST annual variability reflects the larger annual cycle in wind speed in the northern hemisphere, with lower summertime wind speeds^52^. Radio altimeter data from satellite (1986–1995) shows inter-annual variation in wind speed of 7.3 m s^−1^ in the North Atlantic compared with 4.8 m s^−1^ in the Southern Ocean^53^. Summer westerlies in the northern hemisphere mid-latitudes are weaker than their southern hemisphere counterpart due to a larger land fraction, increasing summer temperatures and reducing the poleward temperature gradient^54^. The largest annual cycles arise in semi-enclosed northern hemisphere coastal regions north of the western boundary currents (Bohai Sea, Sea of Japan, Gulf of St. Lawrence) and in enclosed seas (Baltic Sea, Black Sea, Caspian Sea).

In panels 2b to 2d of Fig. 2 we analyse SST anomaly variability on different timescales. In order to do this we first calculate “ten-day” (decad) detrended SSTA composites using fixed daily intervals for each month, defining decads as 1st-10th, 11th-20th and 21st onwards for each month. Data are detrended by removing a linear fit to the data at each location, over the full data record. We use decad averages as a means to reduce observational noise in the SSTA while retaining sub-monthly variability. Some day-to-day real geophysical variability is also thereby suppressed. We remove the linear temperature trend across the full dataset for each data point, and calculate the power spectrum for each location of the detrended SSTA. In Fig. 2b–d we present the SST integrated amplitude over different timescales (the square root of the SSTA variance attributable to different parts of the power spectrum). Figure 2b represents the sub-annual timescale, Fig. 2c a timescale of 1–5 years inclusive and Fig. 2d timescales longer than 5 years.

SST power on sub-annual timescales (Fig. 2b) is greatest in regions characterised by mesoscale eddies, including the major western boundary currents: Gulf Stream, Kuroshio extension, Agulhas current, Brazil-Falkland Confluence and the Falkland current. It is also evident in the equatorial Pacific where SSTA are strongly modulated by TIWs and ENSO variability. The SST power on sub-annual timescales accounts for a maximum variance in SSTA of 4.9 K^2^ in the Brazilian-Falkland confluence, 4.3 K^2^ in the Gulf Stream, 3.5 K^2^ in the Agulhas current, 2.6 K^2^ in the Kuroshio extension and 1.5 K^2^ in the ENSO region. The SST integrated amplitude on sub-annual timescales is also increased in coastal regions, enclosed seas (Black Sea, Caspian Sea), in regions affected by storm tracks in the Southern Hemisphere (~ 30 S) and within Agulhas rings^23^, in comparison with the open ocean.

The SST integrated amplitude over annual to five-year timescales includes slower ocean processes and large-scale ocean modes (Fig. 2c). We see that some SST variability in western boundary currents can be attributed to SST processes operating on those timescales, although this may be aliasing given the relatively short length of the timeseries. In the ENSO region, SST power is dominated by SST processes on 1–5 year timescales, with a maximum SSTA variance of 1.9 K^2^. Processes on this timescale affect a larger area of the equatorial Pacific than sub-annual processes.

Finally we present the SST integrated amplitude from the part of the power spectrum that represents SST processes with a timescale longer than five years (Fig. 2d). The full dataset length of 37 years is relatively short for estimating the SST power in this range, but we can see coherent contributions to SST variability in the ENSO region, the north-east Pacific and the sub-polar gyre south of Greenland, from long-term processes. The SSTA data are detrended using a local linear fit, but in these regions (Fig. 1) we see that the long-term signal is non-linear. This long-term signal in the SST integrated amplitude is likely the residual signal from the detrending process. In the sub-polar gyre we see significant warming in the 1990’s and cooling in the 2010’s whilst the reverse is true for the north-east Pacific in the region of the recent warm anomaly^32^. In the ENSO region the timeseries trend is also non-linear with a cooling in the 1980’s and 1990’s and a warming in the 2010’s following the hiatus period of the 2000’s.

### SST Persistence

Here, we consider how SST anomalies persist across the global oceans. In regions characterised by fast-moving features such as sub-mesoscale eddies, local SSTA persistence can be of order days, whereas in regions where SSTA variability is dominated by large-scale SST modes (eg. ENSO^55^), persistence time scales can be several months. To the first order, the main control on SSTA persistence is the effective heat capacity of the ocean surface determined by the mixed layer depth (MLD)^16,56^. The deeper mixed layer often associated with windy winter conditions in mid-latitudes increases the SSTA persistence^57^. It is also possible to have both short and long-term SSTA persistence occurring in the same location eg. TIWs and Kelvin Wave SST anomalies in the equatorial Pacific (short-term) and the ENSO SSTA signature (long-term).

We attempt to quantify persistence on two time scales. To consider firstly short-term SSTA persistence (<30 days), we quantify this using an exponential decay function^16,57^, fitted to the smoothed auto-correlation timeseries for each location derived from daily detrended SSTA data (please refer to the Methods section for details). We define the SSTA persistence length scale as the number of days before the fitted exponential decay falls below a correlation of 0.6, following Ding and Li^58^. This represents 61% of the e-folding timescale. If the auto-correlation doesn’t fall to 0.6 over a 30-day period, we deem no short-term persistence length is calculable.

**Figure 3:**
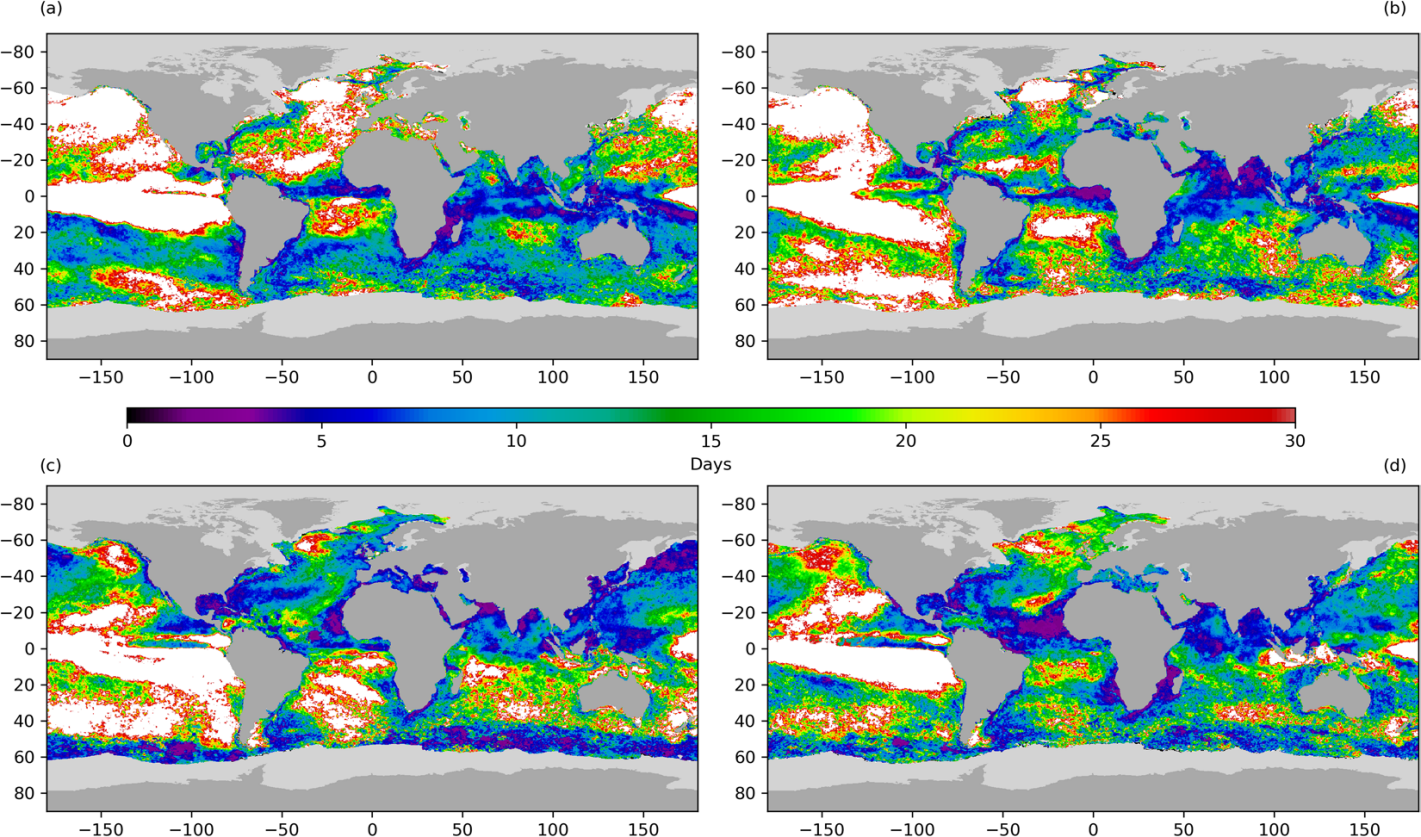
Seasonally-dependent short-term persistence of SST anomalies starting in different months ((**a**) December, (**b**) March, (**c**) June and (**d**) September). Persistence is calculated by correlating SST anomalies from the 1st, 15th and final day of each month with the SST anomalies on subsequent days, over a 30-day period across all years. These daily correlations are fitted using a 15-day Savitzky-Golay filter with the time series truncated where the smoothed data first drop below a correlation of 0.6. An exponential curve fitted to the remaining time series is used to define short-term persistence, using a correlation threshold of 0.6^58^. Ocean regions with no short-term SSTA persistence calculable by this method are coloured white. Sea-ice pixels are shown in light grey, and land in dark grey.

Short-term SSTA persistence has a seasonal dependence as shown in Fig. 3. Here we show SSTA persistence length scales, starting from (a) December, (b) March, (c) June and (d) September. The median global short-term persistence length is 12 days in December, 14 days in March, 11 days in June and 12 days in September. Short-term SSTA persistence is observed over 70.9, 71.0, 71.6 and 78.5% of the global oceans in each season respectively. There are a larger proportion of areas of short-term SSTA persistence observed in the summer hemisphere, when the mixed layer is shallower. Under these conditions new SST anomalies are more readily formed via atmospheric forcing^16^.

Short-term SSTA persistence is prevalent in the tropical and sub-tropical Indian Ocean in all seasons, with time scales typically between 5–10 days. Studies of mesoscale eddies indicate that this is a region characterised by a large seasonal cycle in eddy kinetic energy similar to the western boundary currents^59,60^. In the austral summer and autumn (Fig. 3a,b), we observe SSTA persistence of order 10–15 days across much of the Southern Ocean, particularly in December. Note the absence of short-term persistence results over the sub-polar gyre south of Greenland in boreal winter and spring indicating that SSTAs persist over timescales longer than 30 days here. SSTA persistence is also absent over the north and east Pacific during these months, and parts of the southern Pacific in spring (Fig. 3b). In all seasons we observe no short-term SSTA persistence features over most of the ENSO region (with the exception of propagation of TIWs in the equatorial Pacific), as this is dominated by large-scale SSTA modes.

In the boreal summer and autumn (Fig. 3c,d) we see short-term SSTA persistence associated with the Gulf Stream and Kuroshio extension. The persistence time typically lengthens by a few days in September in comparison with June, consistent with the expected increase in the MLD. In the north and eastern Pacific and sub-polar gyre south of Greenland some short-term SSTA persistence is observed but this is typically in the range of 15–30 days and does not encompass the entire aerial extent of these features. SSTA persistence time is shortest over the tropical Atlantic, western Pacific and equatorial Indian Ocean (typically 5–10 days).

Short-term SSTA persistence is also observed in the equatorial Atlantic, west of Africa, in the regions typically affected by wind-blown Saharan dust. This is most evident in Fig. 3d, but can also be seen across the other seasons (closer to the equator in Fig. 3a,b). This is an artefact of the SST data, where the retrieval is not robust to high concentrations of desert dust. Where dust events are not flagged as cloud in the retrieval, they tend to bias the retrieved SST cold^2,61,62^ and can cause an artificial timescale in the SSTA correlation, which then appears as short-term SSTA persistence.

**Figure 4:**
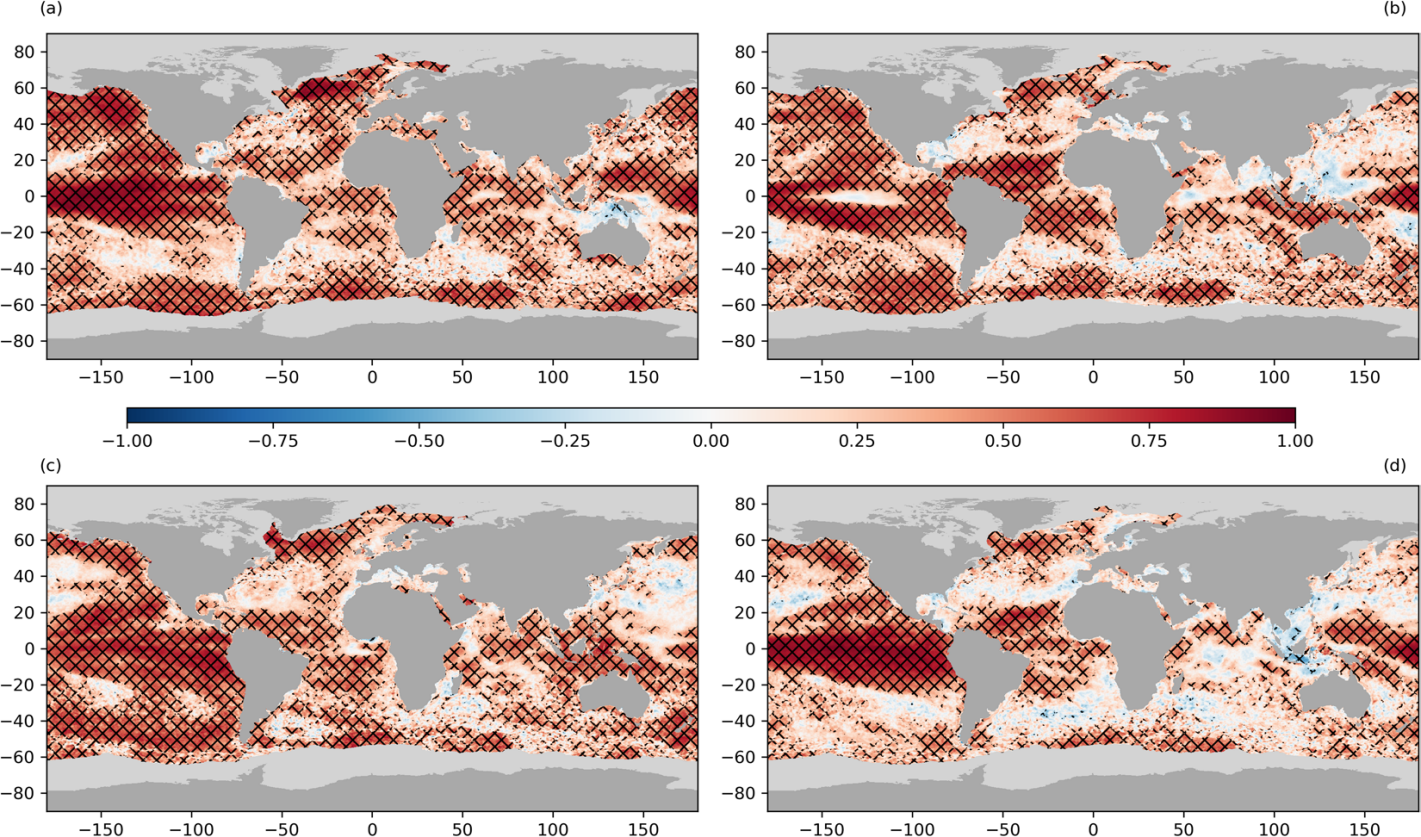
SSTA correlations with a three-month time-lag from (**a**) December, (**b**) March, (**c**) June and (**d**) September. Correlations are calculated for 36 complete years of data (1982–2017). Sea-ice pixels are shown in light grey and land in dark grey. Statistically significant correlations are indicated by the overlaid hatching.

**Figure 5:**
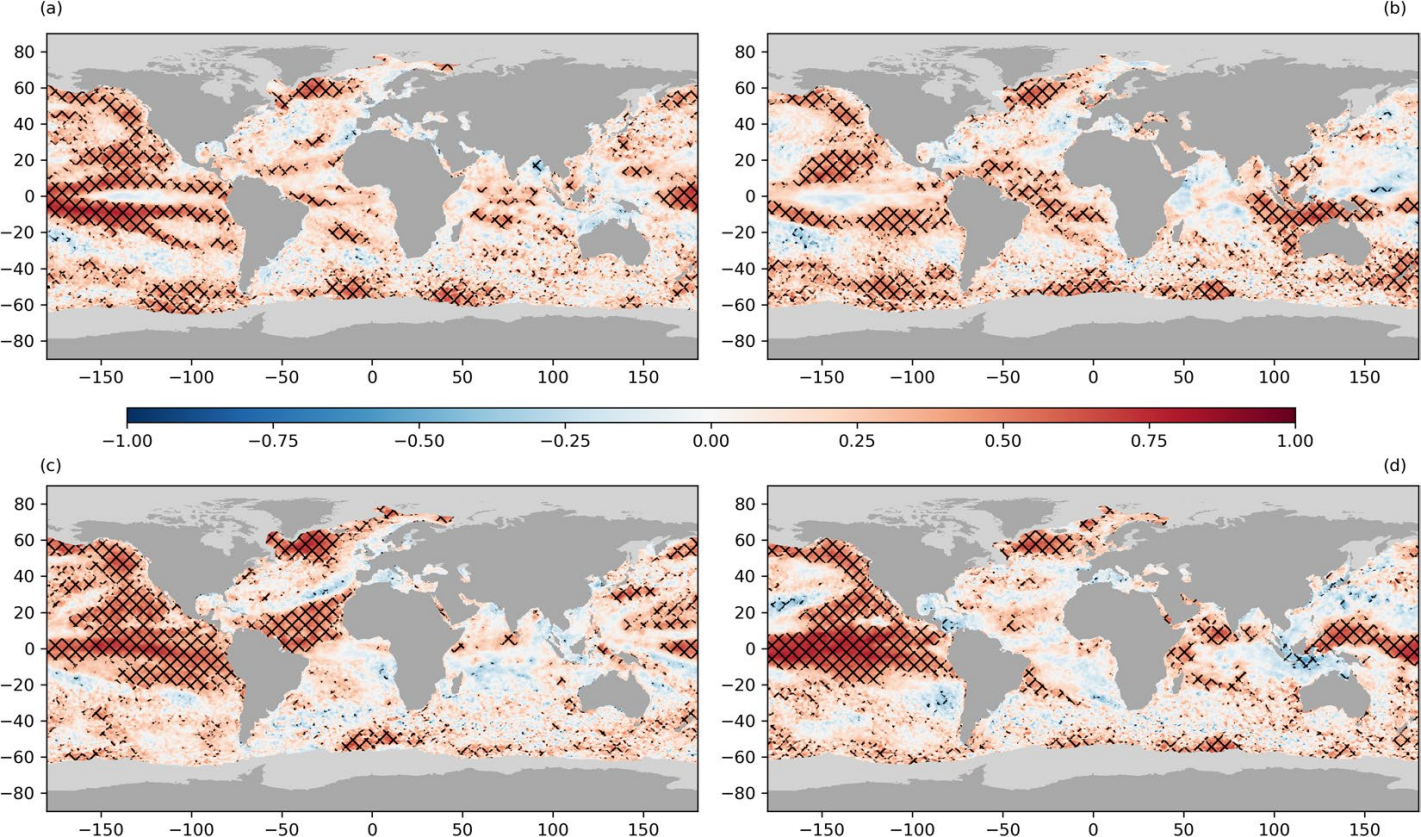
SSTA correlations with a six-month time-lag from (**a**) December, (**b**) March, (**c**) June and (**d**) September. Correlations are calculated for 36 complete years of data (1982–2017). Sea-ice affected pixels are shown in light grey and land in dark grey. Statistically significant correlations are indicated by the overlaid hatching.

**Figure 6:**
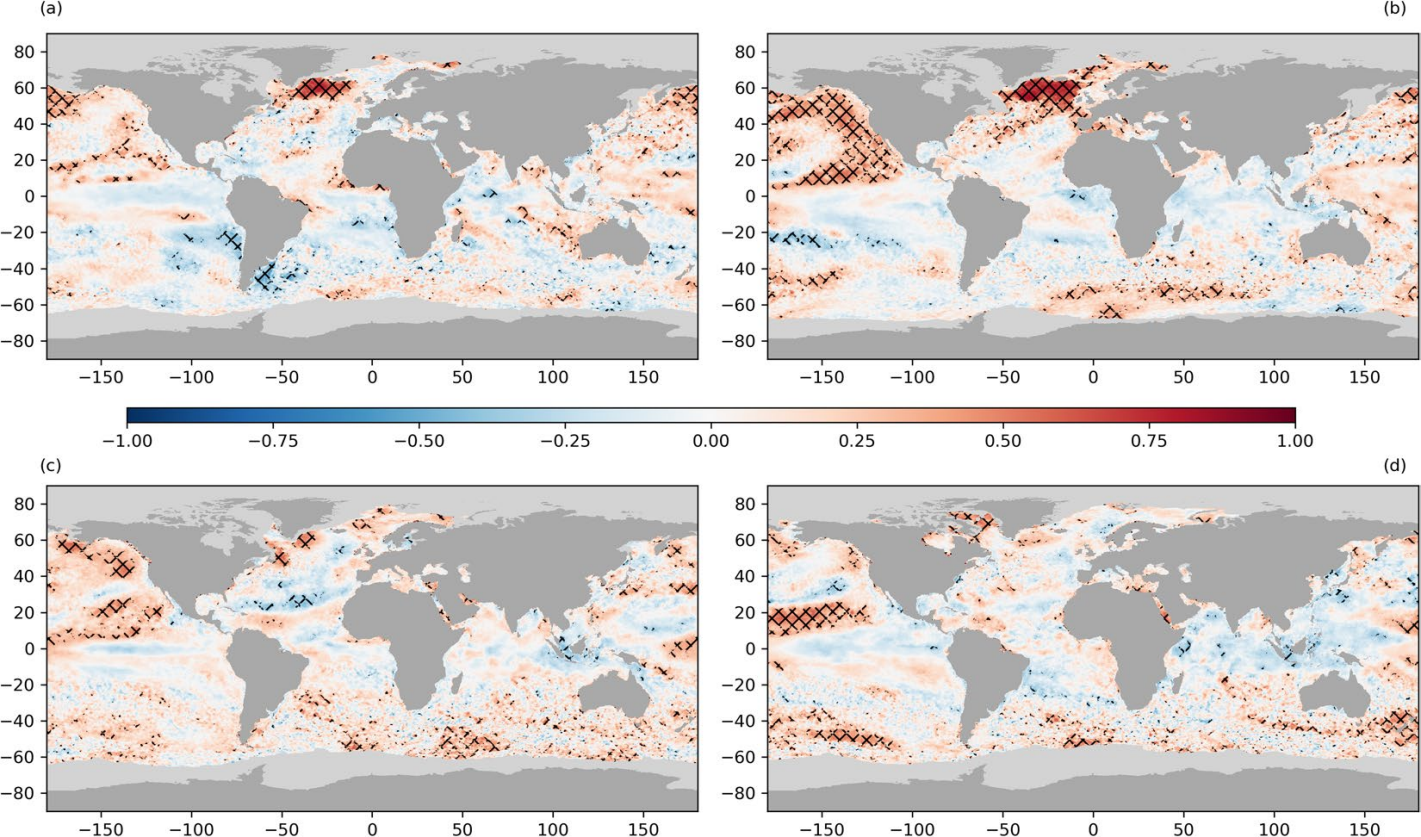
SSTA correlations with a twelve-month time-lag from (**a**) December, (**b**) March, (**c**) June and (**d**) September. Correlations are calculated for 36 complete years of data (1982–2017). Sea-ice affected pixels are shown in light grey and land in dark grey. Statistically significant correlations are indicated by the overlaid hatching.

On longer timescales (up to twelve months) SSTA persistence and re-emergence are coupled. The primary control on both is the mixed layer depth and in regions where this has significant intra-annual variability, re-emergence may be seen. This is typically characterised as winter-to-winter re-emergence although the time lag can be variable, ranging from 7–13 months^15^, and is usually seen in the mid-latitudes^12,15–19^. To quantify the regional and seasonal dependencies of SSTA persistence we present the SSTA correlation at time-lags of three, six and twelve months (Figs. 4–6) for each of the four mid-latitude seasons. The topic of re-emergence is revisited in the discussion section.

Figure 4 shows the detrended SSTA auto-correlation at a three-month time-lag from start months of December, March, June and September. We see statistically significant SSTA correlations (at the 95% confidence level) over 54.1% of the global ocean area (Table 1, averaged over the four seasons). Statistical significance is also shown in Figs. 4–6 by the overlaid hatching). The significance is calculated point-by-point across a data field containing spatial correlation and therefore, for any chosen confidence level, some local areas of statistical significance are expected to arise by chance, which should be kept in mind when interpreting the hatching. The most convincing are the positive correlations found in the ENSO region of the equatorial Pacific, in the sub-polar gyre south of Greenland, in the northern and southern Pacific, parts of the Southern Ocean in boreal winter and in the equatorial and southern Atlantic. These positive correlations in the ENSO region (>0.8) cover the largest area of the Pacific Ocean in the three-month periods from September to December and December to March (austral spring and summer, Fig. 4a,c). Between March and June (Fig. 4b), the positive correlations are weaker and less extensive, particularly within the eastern equatorial Pacific, with the regions of strongest correlation confined to the western and southern equatorial Pacific. Although not strictly phase-locked to the annual cycle, there is an observed tendency for the warm ENSO phase in the Pacific to develop in Boreal summer or autumn and peak in boreal winter^63–65^. El Nino tends to be a single-year event, with equatorial Pacific SST anomalies decaying in boreal spring (March to May)^55,63,65^.

**Table 1:** Mean global area-weighted SSTA correlation for start months of December, March, June and September with time lags of three, six, nine and twelve months.

Start Month	3 Months		6 Months		9 Months		12 Months	
	Correlation	Percentage	Correlation	Percentage	Correlation	Percentage	Correlation	Percentage
December	0.38	59.2	0.19	23.8	0.09	10.4	0.02	6.2
March	0.34	53.8	0.17	23.8	0.1	14.2	0.06	9.5
June	0.37	59.8	0.18	27.0	0.11	16.6	0.06	6.9
September	0.3	43.6	0.18	26.8	0.11	14.6	0.03	7.7

Percentages refer to the percentage of the global ocean area with statistically significant correlations at the 95% threshold.

SSTA correlations in the north-east Pacific and sub-polar gyre south of Greenland with a three-month time-lag are most extensive in winter months (from December, Fig. 4a). In other seasons, although present, the areal extent and strength of this correlation is reduced. SSTAs are positively correlated in the Atlantic tropical gyre for all seasons with a three-month time-lag, with the highest correlations from both March to June and September to December (Fig. 4b,d). In the Indian Ocean south-west of Indonesia positive SSTA correlations are seen with a three-month time-lag from March and from June. The mean global SSTA correlation with a three-month time-lag is 0.35. Global correlations and their statistical significance as a function of both season and time-lag are shown in Table 1.

Figure 5 shows the SSTA correlations with a six-month time-lag. Globally, the correlations are much lower with an average correlation of 0.19, and statistically significant correlations over 25.4% of the global ocean area. The strongest SSTA correlations on this timescale are found in the ENSO region, particularly for the anomalies established in September. For anomaly persistence between December and June we see a reduction in the correlation along the eastern equatorial Pacific (as this time-period covers March to June where the ENSO anomaly breaks down, Fig. 4b). The anomalies from June to December and September to March persist more widely in the ENSO region. We also see statistically significant SSTA persistence south-west of Indonesia between March and September. This breaks down between September and November, corresponding to the time of year where the Indian Ocean Dipole is most likely to transition into its strongest phase as it is phase-locked to the annual cycle^66^. This is consistent with the correlations in Fig. 4, where SSTA are correlated in this region between March and May, and June to August. Statistically significant SSTA persistence is also evident in the sub-polar gyre south of Greenland. These correlations are observed over six-month time periods year round, but are strongest from June to December. We see a weaker SSTA persistence signal in the north Pacific from June to December and September to March, which is statistically significant around the west coast of the United States and northern boundary of the Pacific.

Figure 6 shows the SSTA correlations with a twelve-month time-lag. Statistically significant SSTA correlations occur over the sub-polar gyre south of Greenland in boreal winter, for the start months of December and March. These correlations are strongest and have the greatest aereal extent from March to March. Positive SSTA correlations in the eastern north Pacific from March to March and in the northern hemisphere tropical Pacific from September to September are also statistically significant. These may arise from the significant warming signal in the north Pacific within the 2010s.

## Discussion

**Figure 7:**
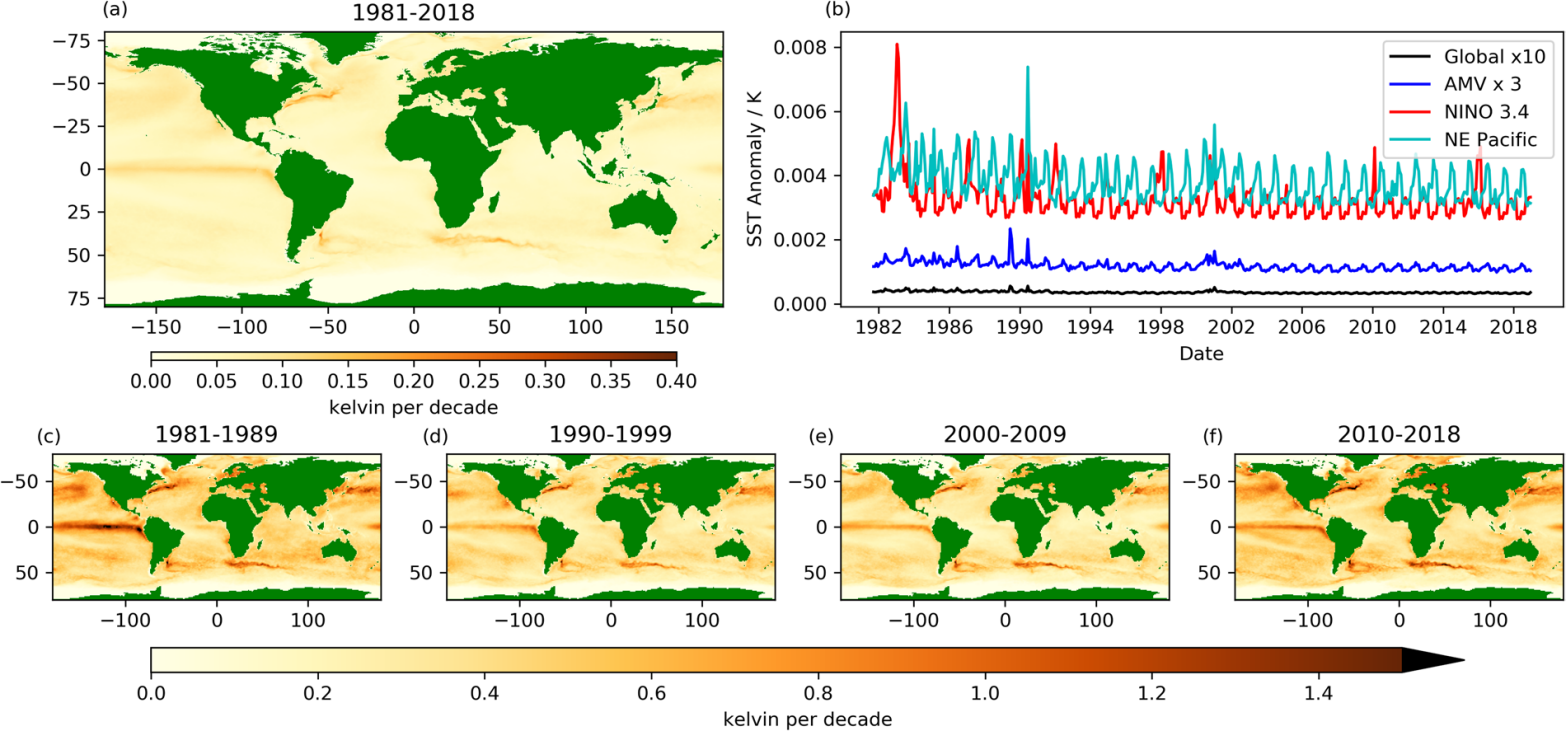
(**a**) Uncertainty in the Theil-Sen slope fit^82^ of the sea-surface temperature anomaly (SSTA) trend between 1981–2018 at 0.25 degree resolution. (**b**) Timeseries of SSTA uncertainties between 1981–2018 for global data (black), the Atlantic Meridional Variability Region [0–65 N, 75–7.5 W^30^ (blue), the Nino 3.4 region [5N-5S, 170–120 W^31^ (red) and the NE Pacific warm waters [32–54 N, 165–138 W^32^ (cyan). Uncertainties in decadal tendencies (degrees kelvin per decade) calculated from data for (**c**) 1981–1989, (**d**) 1990–1999, (**e**) 2000–2009 and (**f**) 2010–2018 are at one degree resolution. Note that the colour scale is different (0–1.5 K) for decadal panels (**c–f**) than for the full-period figure (a, 0–0.4 K).

Interpretation of trends in geophysical variables, particularly in relation to discussions about climate change should take into account the uncertainties inherent in both the data and the trend analysis method. Figure 7 shows aspects of the uncertainties of the data presented in Fig. 1. All uncertainty values are presented as standard uncertainty^67^, sometimes referred to as “one sigma” uncertainty, and approximately corresponding to the 68% confidence interval. In Fig. 7a we show the uncertainty in the fitting of the SSTA trend. The global area-weighted average uncertainty in the local trend fitting (excluding perennial sea ice regions) is 0.043 K. The largest fitting uncertainties occur across the WBCs in both hemispheres and in the equatorial Pacific, which are locations where temperature variability is larger relative to sustained trends. Figure 7c-f show the fitting uncertainties associated with the trends in each decadal plot in Fig. 1c-f. Here we see that the fitting uncertainties are similarly distributed to those for the full timeseries, but are larger as the timeseries are shorter.

Figure 7b shows the analysis uncertainty^68^ in the Level 4 monthly data products used to generate the timeseries data in Fig. 1b. Uncertainties are propagated from the daily, 0.05 degree scale to the monthly, 0.25 degree resolution (for more details refer to the Methods section). The uncertainty values shown do not include the uncertainty from instrument instability over time, which is of the order 0.05 K per decade^69^. Uncertainties are calculated for the global data (excluding regions of perennial sea ice) and for each of the regions examined in Fig. 1b: the AMV, Nino 3.4 region and the north east Pacific. We see a distinct seasonal cycle in the uncertainties, with larger values in the summer hemisphere. This reflects the greater impact of sampling uncertainty in the observations when sub-daily SST variability is increased in summer because of stronger diurnal cycle effects^70^.

There is a general downward trend in the uncertainties from the beginning of the data record until 1995 as satellite data coverage and quality increased. The early 1990’s mark the beginning of the high-quality Along-Track Scanning Radiometer (ATSR) data record. ATSRs introduce SST measurements into the climate data record from sensors specifically designed to stably measure surface temperature. Peaks in the uncertainties in 1982 and 1991 correspond to the major volcanic eruptions of El Chichon and Mount Pinatubo respectively^71^. We also see peaks in the uncertainties in the Nino 3.4 region associated with strong El Nino events in 1982–83, 1991–92, 1997–98, 2000–02 and 2015. The temporary rise in uncertainties in 2000–01 results from instrument issues^2^ (refer to the Methods section for more details on the satellite instruments contributing to the climate data record).

Now considering the trends in the SST data; the cooling in the South Pacific evident in the 1980’s-2000’s (Fig. 1), where historically data coverage is poor^72^, has also been observed in several analysis products derived from *in-situ* observations. Two such datasets; the Extended Reconstructed Sea Surface Temperature (ERSST) and Hadley Centre Sea Ice and Sea Surface Temperature (HadISST) products, are constructed using inputs from the International Comprehensive Ocean-Atmosphere Dataset (ICOADS). ICOADS contains a collection of marine observations from *in-situ* platforms. Data are present in the Southern Ocean from the 1970’s but coverage is incomplete. So in the South Pacific, both ERSST and HadISST products will relax towards the SST prior in the absence of observations. Warming in this region is expected to be much less than the global mean due to the northward advection by Ekman currents of upwelled cold deep-waters^73–75^. However, for a net cooling to occur, an additional forcing is required. Several mechanisms have been proposed to explain this SST cooling including a link with Antarctic sea-ice expansion, which may in part be driven by strengthening westerly winds caused by stratospheric ozone depletion and tropospheric greenhouse gas concentrations^73,75,76^. Fan *et al*.^76^ observed a 0.2–0.4 K cooling per decade in the annual mean SST over the Southern Ocean between 1979–2011 with a corresponding 12% increase in the annual mean Antarctic sea-ice concentration. As climate models in the Coupled Model Intercomparison Project Phase 5 (CMIP5) do not simulate SST cooling in this region or reproduce the suggested mechanisms for SST cooling^76^, this may suggest that natural decadal variability is also important^73,76^, or that the timing of different response mechanisms in the models is not adequately resolved^74,77^. The Pacific Decadal Oscillation (PDO) also transitioned from a positive to negative phase over the 1980’s-2000’s, becoming more neutral in the 2010’s. Cooling in the Southern Pacific is consistent with the PDO becoming more negative (1980’s-2000’s) and warming consistent with a return towards a positive index (2010’s)^35,78^. From the end of 2016 onwards, a sharp decrease in Antarctic sea-ice has also been observed^78^, consistent with a warming trend in the 2010’s. As this only occurs over a small fraction of the time series it does not dominate the long-term trends we see in the satellite era.

Considering the persistence of SST anomalies, it is common practice to represent anomaly decay over time using an exponential function^16,57^. The results are then sensitive to the temporal resolution of the data used, the definition of persistence and the time window over which the exponential fit is made. In recognition of this we calculate SSTA persistence on two different timescales: of order days and of order months. For the longer timescale, re-emergence of SST anomalies can further complicate the definition of persistence. If present, re-emergence will skew an exponential fit to the auto-correlation timeseries, and result in erroneously long SSTA persistence timescales. To avoid this issue, we presented SSTA correlations at different time lags to give clear representation of both anomaly persistence and re-emergence. We consider evidence for re-emergence through comparison of SSTA correlations at different time lags, looking for a decline and then subsequent increase in correlation as evidence of re-emergence. This is most notable in the sub-polar gyre south of Greenland. Larger, positive SSTA correlations are evident with a twelve-month time-lag than at a six-month time-lag (Figs. 5 and 6), when looking at timeseries starting in December and March. This is also evident in March at nine months (not shown). In other regions we either see no clear re-emergence signal or the data are too noisy to identify re-emergence features, probably at least in part due to the limited dataset length (36 years when considering twelve-month time lags). The high spatial resolution of the dataset may also contribute to the difficulty in identifying a re-emergence signal when looking at long-term correlations in a specific location. SSTAs are advected within the ocean over time, and in high-resolution data there is greater likelihood that an anomaly re-emerges in a different resolved location.

## Methods

The SST record used here was created within the European Space Agency (ESA) Sea Surface Temperature (SST) Climate Change Initiative (CCI). The underlying data are the time-series of gap-filled, daily mean SST values of the CCI SST analysis product^2,79,80^. The timeseries spans Sept 1981 - Dec 2018. It was created on a 0.05° grid by combining SST retrievals from the Advanced Very High Resolution Radiometer (AVHRR) and the Along Track Scanning Radiometer (ATSR) series of satellite-borne sensors. The AVHRR data include SST retrieved from AVHRR sensors 7 to 19 inclusive (with the exception of 8 and 10) on-board the National Oceanic and Atmospheric Administration (NOAA) polar orbiting platforms, and the AVHRR on-board the European MetOp-A platform. AVHRR sensors span the entire data record, comprised of Global Area Coverage (GAC) observations with a nominal resolution of 4 km in the nadir. GAC data were included throughout the climate data record (CDR) for consistency, as higher resolution data are not globally available otherwise over that period. ATSR data are from ATSR-1 and ATSR-2 aboard the European Remote Sensing (ERS-1) and ERS-2 satellites and the Advanced Along- Track Scanning Radiometer (AATSR) aboard ENVISAT. These data are at 1 km resolution in the nadir and span the years 1991–2012. For full details of the dataset construction please refer to Merchant *et al*.^2^ and references therein. The grid resolution of the L4 data is 0.05 degrees, with a feature resolution^81^ of the order 20 km resolved within the product. For this work, we use the CCI analysis spatially averaged to 0.25°, thus preserving the SST feature resolution.

We analyse the global trend in the data (Fig. 1) using the full data record (Sept 1981- Dec 2018). Sea surface temperature anomalies are calculated with respect to a daily climatology, constructed using all data for a given day between 1982 and 2010. We plot the trend in the global data at monthly resolution, applying a binomial weighting function (spanning six months on either side of each observation) to produce the smoothed data in Fig. 1b. At the edges of the time series the width of the binomial weighting is reduced appropriately. The same methodology is applied to the anomaly time series for the extracted regions with only the smoothed data shown. The remaining analysis uses the detrended timeseries. We calculate decad SSTA products (10-day averages) using fixed intervals for each calendar month (1st-10th, 11th-20th, 21st onwards). Using decad products increases the temporal resolution of the data over monthly averages, resolving mesoscale features whilst ensuring independence by recognising the temporal error correlation in the analysis product; of order 3 days due to the analysis observation window. The decad SSTA products are then detrended by removing the linear fit to the monthly timeseries in each location. This linear fit is calculated using the Theil-Sen estimator based on Sen^82^, which calculates an unbiased regression on the basis of Kendall’s rank correlation. From this detrended timeseries we calculate the SSTA power spectra and analyse the SSTA variance that can be attributed to processes operating on different timescales. We present the integrated amplitude (square root of this variance) in Fig. 2 to more clearly show the SSTA variability, on sub-annual, one to five year and longer than five year timescales.

Short-term SSTA persistence (Fig. 3) is calculated by correlating the SST anomaly on a given day (T_1_) with the SST anomaly on subsequent days (*T*_2,_*T*_3_…*T*_*n*_). Considering seasonal variations, for each start month (Dec, Mar, Jun and Sept) we correlate the first 30 days, expanding our sample by using three start dates in each of these months (1st, 15th and last day of the month) across all of the years. We fit a Savitzky-Golay filter^83^ with width 15 days and polynomial order three to each correlation timeseries, which fits a localised cubic function by least squares regression, removing the remaining noise without losing the large-scale time-dependent trend in the correlation. We threshold this filter at a correlation of *r* = 0.6 to define the dataset length and fit an exponential decay curve to these data. The persistence is defined as the point at which this exponential curve crosses the correlation of 0.6, consistent with Ding and Li^58^.

To examine long-term SSTA persistence and re-emergence we plot the correlation coefficient for SST anomalies with different time-lags, in each mid-latitude season. We choose start months of December, March, June and September to represent the different seasons, and consider time-lags of three, six and twelve months. For any given month, we take the monthly average SSTA for each year of the dataset and correlate this with the monthly average at the given time-lag, using a Pearson correlation. Each correlation coefficient is calculated using a total of 36 data points, as the final year is excluded from the list of start months in order to be able to consider a time lag of up to twelve months for each starting location.

The uncertainty analysis in Fig. 7 has two components. The uncertainty in the Theil-Sen slopes used to describe the SST trend over the period of the dataset is calculated using a one sigma confidence interval, which also follows the methodology of Kendall’s rank correlation based on the median of the set of slopes between each pair of points in the regression^82^. The uncertainties in Fig. 7b are propagated from the analysis uncertainties from the Level 4 SST product calculated using optimal interpolation^68^. These uncertainties are designed to indicate the weight of observations in the analysis convolved with the background error variance estimate^68^. We average the uncertainty when regridding the daily data from 0.05 degrees to a 0.25 degree product (assuming that these data are fully correlated over this spatial scale). Uncertainties are then propagated assuming a temporal correlation length scale of 3 days and a spatial correlation of 100 km. The latter is consistent with the larger spatial correlation length scale assumed in the Level 4 optimal interpolation^68^.

## Data Availability

The European Space Agency (ESA) Climate Change Initiative (CCI) Sea Surface Temperature data used in this publication are freely and publicly available. The dataset has the following  10.5285/62c0f97b1eac4e0197a674870afe1ee6 and can be accessed via http://data.ceda.ac.uk/neodc/esacci/sst/data/CDR_v2/Analysis/L4/v2.1.
